# External Fixator‐assisted Ulnar Osteotomy: A Novel Technique to Treat Missed Monteggia Fracture in Children

**DOI:** 10.1111/os.12426

**Published:** 2019-02-04

**Authors:** Qiang Wang, Meng‐meng Du, Xin‐jian Pei, Jun‐zhong Luo, Ya‐zhou Li, Yu‐chang Liu, Xuan Wang, Jin‐chao Cao, Jiu‐hui Han

**Affiliations:** ^1^ Department of Pediatric Orthopaedics The Third Hospital of Hebei Medical University Shijiazhuang China

**Keywords:** External fixator, Missed, Monteggia fracture, Ulnar osteotomy

## Abstract

**Objective:**

The treatment of missed Monteggia fracture remains a challenge, despite the various surgical methods described. The purpose of this study was to explore a new surgical technique utilizing external fixator‐assisted ulnar osteotomy and to assess the surgical results in a case series.

**Methods:**

Thirteen patients with missed Monteggia fractures were treated at our institution using this new surgical technique from August 2012 to January 2016. Our series included 11 boys and 2 girls. The left elbow was involved in 6 patients and the right elbow was involved in 7 patients. According to the Bado classification, 10 fractures were classified as Bado type I with anterior radial head dislocation and 3 were classified as Bado type III with anterolateral dislocation. The average age at the time of surgery was 5 years 8 months (range, 2 years 2 months–10 years). The mean trauma‐to‐surgery interval was 12 months (range, 2–36 months). All patients underwent ulnar osteotomy with angulation and lengthening using a temporary external fixator, plate fixation of the osteotomy, and open reduction of the radial head dislocation without annular ligament reconstruction.

**Results:**

The average follow‐up was 27 months (range, 16–44 months). The average operation time was 175 min (range, 140–215 min). The average length of distraction was 0.7 cm (range, 0.5–1.2 cm) and the average angulation was 28° (range, 20°–30°) at the ulnar osteotomy site intraoperatively. The elbow performance score (Kim's) was excellent in 10 cases and good in 3 cases. No neurovascular complications, compartment syndrome or implant breakage occurred. No pain in the distal radioulnar joint or limited range of motion of the wrist occurred in any patient. The radial head remained reduced in all patients with no subluxation or redislocation. However, delayed ulnar union occurred in 3 cases, all of which were successfully treated with plaster cast immobilization within approximately 6 months postoperatively. One patient presented with cubitus valgus postoperatively with a carrying angle of 30°, which was 10° greater than the contralateral carrying angle.

**Conclusions:**

External fixator‐assisted ulnar osteotomy offers substantial flexibility for achieving the optimal positioning of the transected ulna to reduce the radial head prior to the final ulnar osteotomy fixation with a plate, thereby facilitating an effective operative performance. Our procedure is a safe and effective method to treat missed pediatric Monteggia fractures.

## Introduction

Monteggia fractures originally referred to fractures of the shaft of the ulna accompanied by anterior dislocation of the radial head, as described by the Italian surgeon Giovanni Battista Monteggia in 1814. Subsequently, Jose Luis Bado classified Monteggia injuries, which included type I to IV according to the direction of dislocation of the radial head and angulation of ulna shaft fracture, in 1958. Type I is the most frequently seen type in children, accounting for approximately 70% of all suchlesions^1^. A Monteggia fracture is a relatively rare injury, occurring in 1% of all pediatric forearm fractures^2^. However, it is also one of the most frequently missed injuries. In patients with plastic deformation or in whom the physician's emphasis is misdirected towards the ulnar displacement, radial injury is often overlooked. This oversight is further compounded by poor‐quality imaging of the elbow. Babb *et al*. report that approximately 16%–33% of these injuries might be missed initially^3^. A missed Monteggia lesion is defined as a radial head dislocation that is not reduced and remains present 4 weeks after injury^4^, ^5^. The natural history of missed Monteggia lesions is associated with increasing valgus deformity and elbow disability, causing pain, decreased range of motion, overgrowth of the radial head, and late ulnar nerve palsy, which are unacceptable sequelae in pediatric patients, and can be expected when the injury is left untreated^6^, ^7^, ^8^.

The treatment of a missed Monteggia fracture remains challenging, as reflected by the numerous surgical procedures described, including ulnar osteotomy, open reduction of the radial head dislocation with or without annular ligament reconstruction (ALR), radial osteotomy, and radial head excision at the end of the growth^4^, ^5^, ^7^, ^8^, ^9^, ^10^. In patients with missed Monteggia lesions, the normal anatomical relationship between the radius and the ulna is lost. The deformity of the ulna is the main obstacle to reduce the radial head and to maintain the reduced radial head in position. Therefore, ulnar corrective osteotomy to restore the ulnar axis and ulnar length is the key procedure of the surgery, and lengthening and angulation in the ulnar osteotomy site are the most important surgical procedures^5^, ^7^, ^8^, ^9^. Sometimes, tightening of the interosseous membrane of the forearm through over ulnar angulation is needed to maintain the reduction. Judet *et al*. first reported the use of corrective osteotomy of the ulna for the treatment of dislocation of the radial head in missed Monteggia fractures in 1962. Ulnar osteotomy was universally accepted after Hirayama *et al*. published their results with open reduction and ulnar osteotomy for missed Monteggia lesions^11^. Then various modifications of this technique were developed, but the outcome was not uniform with reports of subluxation and redislocation as well as complications, including pain, stiffness, elbow instability, cubitus valgus deformity, nonunion of the osteotomy, avascular necrosis of the radial head, and nerve injury^4^, ^5^, ^6^, ^7^, ^8^, ^9^, ^10^. After osteotomy, plates were used to directly fix the lengthened and angulated ulna in most reported cases^4^, ^5^, ^6^, ^8^, ^9^. However, maintaining the position of the lengthened and angled osteotomy ends when placing the plates intraoperative is difficult; if the radial head dislocates during the stability test, further opening, readjusting of the osteotomy ends, and repeat plate placing are required, which makes the surgeries more complicated.

To facilitate ulnar distraction and angulation and to allow assessment of radiocapitellar joint stability before final ulnar fixation, we applied a new technique to treat missed Monteggia fractures using external fixator‐assisted ulnar lengthening and angulation intraoperatively prior to final ulnar fixation using a plate. ALR was not performed in any of the cases in our study. From August 2012 to January 2016, 13 cases of missed Monteggia fracture were managed with this new technique. The purpose of our retrospective study was to review the clinical outcomes of patients treated using this specific technique of ulnar osteotomy.

## Materials and Methods

We performed a retrospective review of 13 cases of missed Monteggia fracture treated at the Third Hospital of Hebei Medical University from August 2012 to January 2016. The surgical strategy for all patients included temporary external fixator‐assisted ulnar osteotomy, open reduction of the radial head dislocation, and ulnar osteotomy plate fixation. No patients underwent ALR. We used the Boyd approach (3 cases) and the Henry approach with proximal ulnar dorsal incision (10 cases).

Our series included 11 boys and 2 girls. The left elbow was involved in 6 patients and the right elbow was involved in 7 patients. According to the Bado classification, 10 fractures were classified as Bado type I with anterior radial head dislocation and 3 were classified as Bado type III with anterolateral dislocation. The average age at the time of surgery was 5 years 8 months (range, 2 years 2 months–10 years). The mean trauma‐to‐surgery interval was 12 months (range, 2–36 months). One patient presented with radial nerve palsy, two had cubitus valgus, and two exhibited limitation of flexion before surgery. At follow‐up, clinical physical examinations and X‐rays, including an evaluation of elbow motion, deformity and stability, were performed. All intraoperative and postoperative complications were recorded. Function was evaluated using Kim's elbow performance score^12^, which assesses pain, deformity, range of motion and function (scored between 0 for worst and 100 for optimal). The total scores were categorized as excellent (>90), good (89–75), fair (74–60), and poor (<60).

### *Surgical Technique*

**Figure 1:**
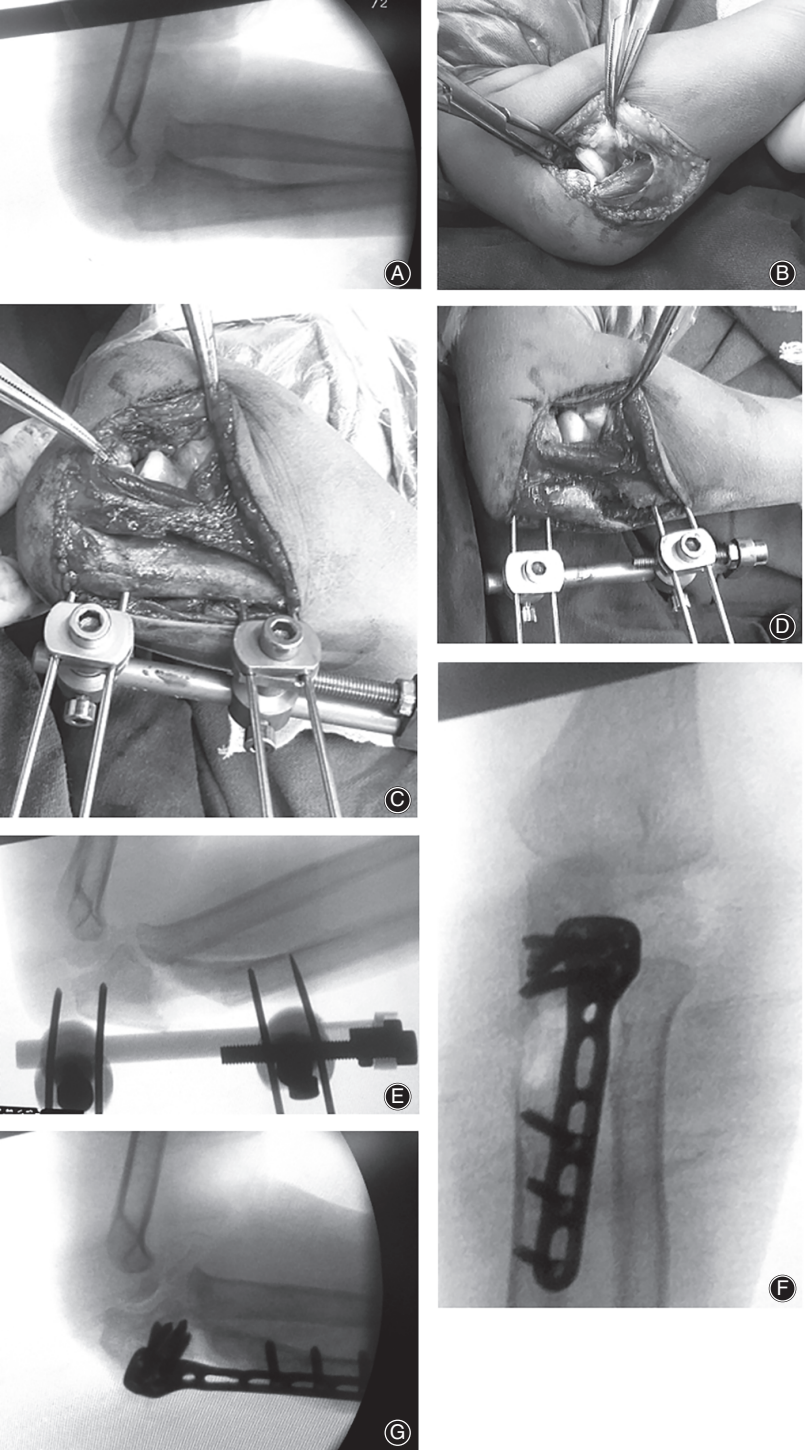
Surgical diagram of external fixator‐assisted ulnar osteotomy. (A) Lateral radiographs showing a missed Bado I Monteggia fracture in a 6‐year‐old child. (B) Intraoperative photograph demonstrating anterior dislocation of the radial head. (C) An external fixator is placed on the proximal ulna dorsally before osteotomy. (D) An external fixator‐assisted ulnar lengthening and angulation at the osteotomy site facilitated reduction of the radial head. (E) Intraoperative fluoroscopic picture showing the ulnar osteotomy and reduction of the radial head. (F, G) Anteroposterior and lateral radiographs showing the final fixation with a plate and complete restoration of the radiocapitellar joint.

The Boyd approach or the Henry approach was used to expose the radiocapitellar joint. The radiocapitellar joint was debrided of any residual fibrous scar tissue that hindered radial head reduction. The ulna was exposed, and a unilateral external fixator was applied to the ulna using 2 proximal and 2 distal Kirschner wires (2 mm), ensuring placement of the proximal Kirschner wires as close to the coronoid process as possible to facilitate the proximal ulnar osteotomy (Fig. 1A–C). An oblique osteotomy of the ulna was then performed. The osteotomy site was distracted with posterior angulation for Bado type I cases and with posterior and medial angulation for Bado III cases to overcorrect the ulnar deformity using the external fixator and, thus, reduce the radial head (Fig. 1D,E). The degrees of angulation and distraction were determined by evaluating the stability of the radial head reduction. We tested the stability of the radiocapitellar joint by pronating the forearm in a 90° flexed elbow position; if the radial head dislocated during pronation, it was considered unstable, and further angulation and lengthening of the ulnar osteotomy were performed by adjusting the external fixator until a stable position was achieved in both full supination and pronation. When stable radial head reduction was achieved, the ulna was fixed using a pre‐bent Φ2.7‐mm locking compression plate (Zheng Tian Medical Instrument, Tianjin, China) (Fig. 1F,G). Allograft bone graft substitute (OsteoRad Biomaterial, Shanxi, China) was used in all cases. The joint capsule was tightened using absorbable sutures.

In 1 case (case 2) in which the interval before surgery was longer than 3 years, the radial head was unstable when the forearm was placed in extreme pronation, regardless of the intraoperative fixator adjustments. Therefore, a Kirschner wire was used to fix the radiocapitellar joint. Postoperatively, a long‐arm plaster splint with the elbow in 90° flexion and the forearm in supination was applied in all cases and was removed 6 weeks after surgery in most cases. After splint removal, active forearm rotation and flexion and extension of the elbow were initiated.

## Results

### *General Results*

The mean follow‐up duration was 27 months (range, 16–44 months). The average operation time was 175 min (range, 140–215 min). One patient presented with radial nerve palsy preoperatively, which showed complete recovery 3 months after surgery.

### *Radiological Results*

The average length of distraction was 0.7 cm (range, 0.5–1.2 cm) and the average angulation was 28° (range, 20°–30°) at the ulnar osteotomy site intraoperatively. The mean duration of radiological union was approximately 8 weeks in 10 patients. Three patients (cases 7, 10 and 11) had delayed union and eventually achieved union with plaster cast immobilization at approximately 6 months postoperatively. The radial head remained reduced in all patients, and no subluxation or re‐dislocation was observed during follow‐up.

### *Functional Results*

No patients had rotational limitation, but patients 7 and 10 exhibited limitation of extension at the final follow‐up. One patient (case 2) who had undergone radiocapitellar pinning during surgery also showed limited extension during follow‐up. No pain in the distal radioulnar joint or limited range of motion of the wrist occurred in any patient. Kim's score was excellent in 10 cases and good in 3 cases.

### *Complications*

No wound infections, compartment syndrome, neurovascular complications or implant breakage occurred. One patient exhibited skin irritation because of a protruding plate end, which resolved after the plate was removed. One patient presented with cubitus valgus postoperatively with a carrying angle of 30°, which was 10° greater than the contralateral carrying angle (patient 10). Two patients (patients 2 and 7) who had cubitus valgus preoperatively showed no change in their carrying angles postoperatively. The patient outcomes are shown in Table 1.

**Table 1:** Procedures for each patient

Case no.	Sex	Age at Operation (y.mo)	Trauma‐to‐surgery interval (mo)	Ulnar lengthening at osteotomy site (cm)	Ulnar angulation at osteotomy site (°)	Follow‐up (mo)	Postoperative complications	Additional treatment	Kim score
1	M	4	8	0.5	30	36	–	–	100
2	F	4.8	36	0.5	30	24	Loss of extension (20°)	K‐wire fixing	80
3	M	3.11	2	0.7	30	43	–	–	95
4	M	8	2	0.5	20	29	–	–	100
5	M	7	4	0.6	25	25	–	–	95
6	M	5	12	0.8	30	19	–	–	100
7	M	7	3	1.2	30	17	Delayed union 5 mo healed. Loss of extension (35°)	Plaster casts	85
8	F	2.2	13	0.6	25	38	–	–	100
9	M	4.4	17	0.7	30	16	–	–	95
10	M	5	27	1.2	30	20	Delayed union 6 mo healed. Loss of extension (30°) Cubitus valgus (30°)	Plaster casts	80
11	M	7	4	0.8	25	17	Skin irritation. Delayed union 6 mo healed.	Plaster casts	95
12	M	10	9	0.7	30	44	–	–	100
13	M	7	15	0.8	25	24	–	–	90
Mean	–	5.8	12	0.7	28	27	–	–	93

F, female; M, male; mo, months; y, years.

### *Typical Case*

**Figure 2:**
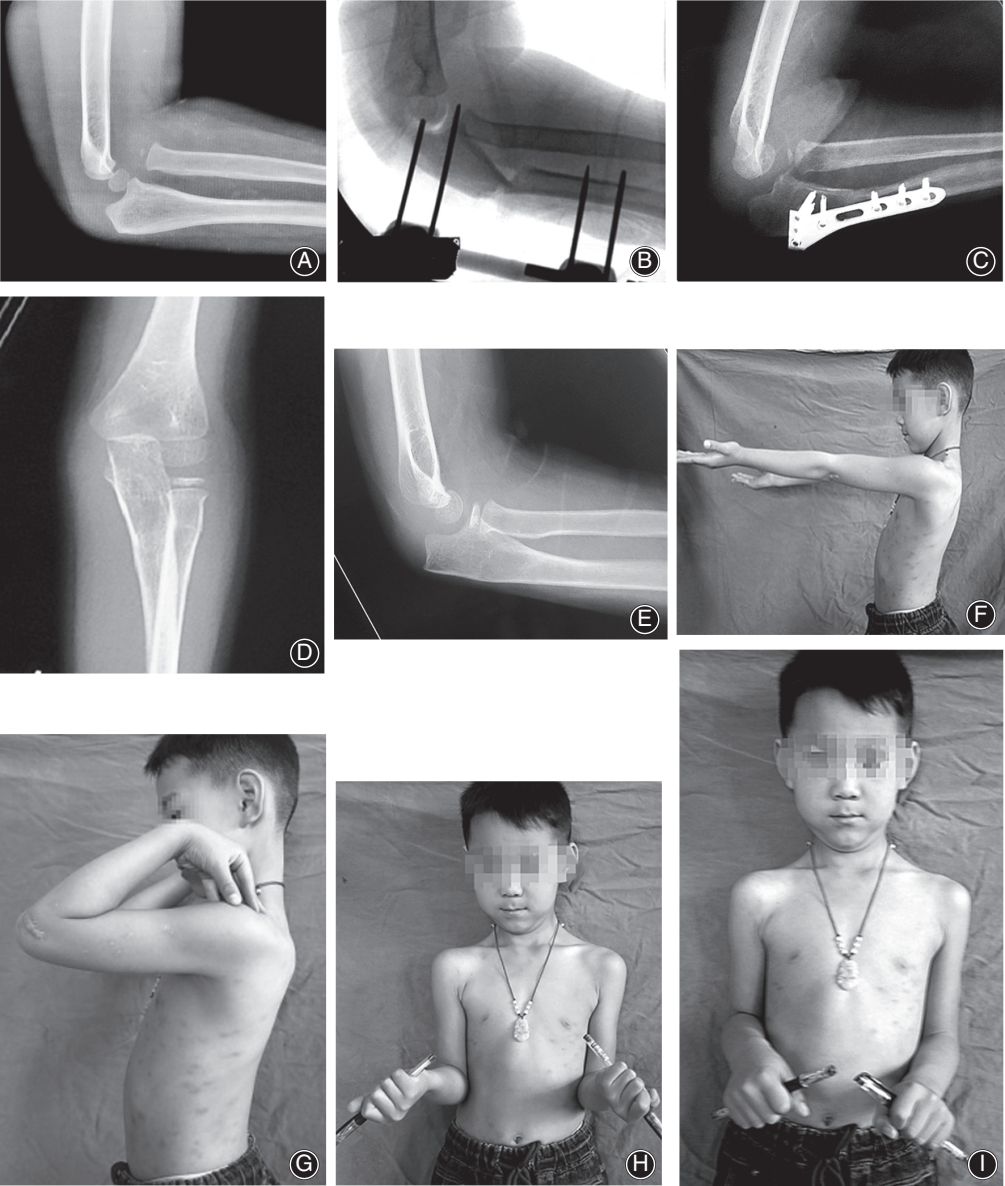
A 4‐year‐old boy fell to the ground and injured his left elbow 8 months before surgery. (A) Preoperative lateral radiograph of the elbow showing a missed Bado I Monteggia fracture. (B) Intraoperative fluoroscopic picture of ulna osteotomy with angulation and lengthening assisted by an external fixator. (C) Lateral radiograph of the elbow showing good remodeling at the osteotomy site at 6 months postoperatively before removing the plate. (D, E) Anteroposterior and lateral radiographs of the left elbow showing a well‐contained radial head at 3‐year follow‐up. (F–I) The clinical examination disclosing full range of motion of the elbow flexion (F), extension (G), supination (H), and pronation (I) at 3‐year follow‐up.

A 4‐year‐old boy (case 1) fell to the ground, injured his left elbow 8 months before surgery and sustained a type I Bado fracture (Fig. 2A). Physical examination revealed no limited range of motion in his left elbow. The surgical strategy consisted of temporary external fixator‐assisted ulnar osteotomy, open reduction of the radial head dislocation, and plate fixation of the ulnar osteotomy. The length of distraction was 0.5 cm and the angulation was 30° at the ulnar osteotomy site during the operation (Fig. 2B). Postoperatively, a long‐arm plaster splint was applied for 6 weeks and then removed. The boy was encouraged to begin moving the elbow and returned to normal activities within 1 month. At 6 months postoperatively, the osteotomy site showed sufficient remodeling, and the plate was removed (Fig. 2C). At the 3‐year follow‐up, the radial head remained reduced (Fig. 2D,E), and the clinical examination showed full elbow range of motion (Fig. 2F–I).

## Discussion

For pediatric patients with missed Monteggia fractures, surgical treatments are often required to achieve and maintain an anatomically normal radiocapitellar joint and, thus, prevent further degenerative joint changes. Multiple treatment strategies have been proposed for this demanding condition, and radial head dislocation and subluxation are the most common complications^4^, ^9^, ^10^, ^11^, ^12^, ^13^. Among all the described surgical procedures, most include ulnar osteotomy with lengthening and angulation. This approach is based on the concepts that malunion of the ulna prevents reduction of the radial head and that tightening of the interosseous membrane through angulation can return the radial head to an acceptable position. After osteotomy, plates were used to directly fix the lengthened and angulated ulna in most reported cases^4^, ^5^, ^6^, ^8^, ^9^. One challenge of direct fixation with plates is that if a longer length and a greater angle of the ulna are required to reduce the radial head, then maintaining the position of the osteotomy ends when placing the plates intraoperatively is difficult. Another challenge is that the stability test, which is critical for surgical success, can be performed only after plate fixation. If the radial head dislocates during the stability test, further opening of the osteotomy is required, and the ulna length and angle are readjusted until a stable position is achieved^7^, ^13^. However, repeatedly removing and placing fixation hardware intraoperatively will prolong the operation and, more importantly, might affect the bone quality at the osteotomy sites, which will degrade the stability of the final fixation.

We applied a new technique using temporary fixation with an external fixator, which facilitated ulnar distraction and angulation and allowed assessment of radiocapitellar stability before final ulnar fixation. In addition, temporary fixation using an external fixator allows substantial flexibility in adjusting the angulation and length of the osteotomy and simplifies the final plate fixation. Due to the advantages of this technique, no complications of radial head dislocation or subluxation were observed in our series.

Several studies have described using external fixation continuously for the ulnar osteotomy site until bony union occurs^10^, ^12^, ^14^, ^15^, ^16^. The most frequently expected complication with the use of external fixation is pin‐tract infection, particularly in delayed union cases with long‐term external fixation, which might lead to mechanical pin loosening and, ultimately, cause instability of the external fixator pin–bone construct^17^, ^18^. All our osteotomies were ultimately fixed with plates to obtain rigid fixation, to facilitate postoperative care, and to mitigate the complications described above. Therefore, our method provides the dual advantages of the flexibility of an external fixator and the stability of internal fixation.

We opened the radiocapitellar joint in every case. Dense fibrous scar tissue over the radial head and the joint was common in these cases, and required excision to achieve reduction of the radial head. However, open reduction of the joints is controversial. Lädermann *et al*. relied only on ulnar osteotomy for reduction of the radial head^19^. Exner *et al*. report that after gradual lengthening of the ulna with an external fixator, reduction of the radial head is possible without radiocapitellar joint opening^16^. Therefore, ulnar osteotomy alone without joint opening might be attempted in very early cases in the future, particularly for patients with a 2‐month trauma‐to‐surgery interval.

No patients exhibited rotational limitation at the final follow up in our group, even with overcorrection of the ulna, which was similar to the results of Inoue^20^. Several authors have reported that ALR results in limited forearm rotation, particularly pronation^4^, ^21^. Other complications related to ALR include elbow stiffness and heterotopic ossification^22^, ^23^, ^24^. Rahbek *et al*. compared the results of 10 patients who underwent ligament reconstruction with ulnar osteotomy with those of 6 patients who underwent ulnar osteotomy alone and found no significant differences in radiographic or clinical outcomes^13^. ALR was not performed in any of the cases in our study.

In our series, we observed 3 cases of delayed ulnar union. Delayed healing might be caused by excessive lengthening of the ulna, which was 12 mm in 2 cases (patients 7 and 10). Lu also reported delayed union due to the same reason^12^. A lengthening of 10 mm was recommended by Hirayama^11^. Another factor responsible for delayed union could have been unstable fixation of the long‐arm splints in our study. We suggest that if lengthening of more than 10 mm is required for radial head reduction, an autogenous bone graft should be applied, and a plaster cast should be used postoperatively. Gradual ulnar lengthening with external fixation or radial shortening might be other alternatives in cases in which a more than 10‐mm ulnar length extension is required.

Our study has limitations. First, the cohort was small. The data referenced regarding chronic Monteggia fractures are only available from case reports or small case series^9^, ^10^, ^11^, ^12^, ^14^, ^15^. The largest chronic Monteggia lesion series were those of Delpont *et al*.^25^, with 28 cases, and Tajima and Yoshizu, with 23 cases^26^. A larger study would help validate our results. The second limitation is that the study design was retrospective, resulting in an intrinsic follow‐up bias. The third limitation was the absence of a control group of patients who underwent traditional plate fixation of the ulnar osteotomy for comparison with our patients who underwent temporary external fixation.

In conclusion, this technique offers substantial flexibility in the optimal positioning of the ulnar osteotomy to achieve stable radial head reduction prior to final fixation. Therefore, this approach is convenient and exhibits effective operative performance, and no complications of radial head dislocation or subluxation were observed in our series.

## Author Contributions

J.H. is the guarantor of the integrity of the entire study. J.H. and Q.W. performed the study concepts/design. Q.W., M.D., X.P., and J.H. performed the data acquisition and/or data analysis. Y.L.,Y.L., J.L., and Q.W. performed the drafting and critical revision of the manuscript. All the authors read and approved the final manuscript. Y.L. and J.L. performed the literature research. J.H., J.C., and X.W. edited the manuscript.
